# Target estimands for efficient decision making: Response to comments on “Assessing the performance of population adjustment methods for anchored indirect comparisons: A simulation study”

**DOI:** 10.1002/sim.8965

**Published:** 2021-05-08

**Authors:** David M. Phillippo, Sofia Dias, Anthony E. Ades, Nicky J. Welton

**Affiliations:** ^1^ Bristol Medical School (Population Health Sciences) University of Bristol Bristol UK; ^2^ Centre for Reviews and Dissemination University of York York UK

1

We thank Remiro‐Azócar, Heath, and Baio (R‐AHB) for their letter to the editor,^1^ in response to our recent article presenting a simulation study comparing the performance of methods for population‐adjusted indirect comparison.^2^ R‐AHB discuss the important issue of target estimands with noncollapsible effect measures, expanding upon the discussion in sections 4.3 and 7 of our article.^2^ R‐AHB distinguish between marginal and conditional treatment effect estimates and explain that matching‐adjusted indirect comparison (MAIC) targets marginal effects whereas simulated treatment comparison (STC) and multilevel network meta‐regression (ML‐NMR) target conditional treatment effects. They conclude that “methods like MAIC are valid for population‐based inference, but not “fit for purpose” when inference is at the individual level, whereas methods like ML‐NMR are valid for inference at the individual level, but not designed for population‐based inference.” Furthermore, they assert that marginal treatment effect estimates are necessary for population‐based inference as required for decision‐making in Health Technology Assessment (HTA).

We welcome and encourage debate of these issues, which—despite much discussion in the literature on randomized controlled trials (RCTs)^3^, ^4^, ^5^, ^6^ and observational epidemiology^7^, ^8^, ^9^—have largely been overlooked in the literature on population adjustment and meta‐analysis to date. However, whilst we agree with R‐AHB that population‐based inference is required for HTA, we disagree that methods like ML‐NMR are not appropriate to obtain population‐average estimates for HTA. In this response, we further clarify the use of conditional estimates to inform population‐average treatment effects and why we believe these are appropriate target estimands for decision making. We also correct some important inaccuracies in R‐AHB's letter regarding the characterization of the methods (in particular ML‐NMR) and interpretation of our simulation study results.

## 2 TARGET ESTIMANDS FOR DECISION MAKING

Healthcare decision making requires estimates of average relative treatment effects between each treatment of interest in the decision target population. Ideally, such estimates would be provided by a well‐designed, representative RCT comparing all treatments of interest in the decision target population. To help solidify ideas and define terminology, let us first consider the analysis of this ideal RCT.

The simplest analysis of this ideal RCT is an unadjusted analysis (eg,  ANOVA or regression including only the main effect of treatment). However, this is not the most efficient approach. Instead, it is recommended practice to include prespecified prognostic factors in the analysis model (eg,  ANCOVA or regression including main effects of covariates and treatment).^3^, ^4^, ^5^, ^6^, ^10^ The adjusted analysis is more powerful and more efficient because some of the additional variation in the outcome not due to treatment has been conditioned on the covariates.^3^, ^4^, ^5^, ^6^ The unadjusted analysis results in *marginal* treatment effect estimates, whereas the adjusted analysis results in *conditional* treatment effect estimates. Since only main effects of covariates and no interactions with treatment have been included in the adjusted analysis, the conditional treatment effects apply over the entire study population; they do not vary by covariate values and are not subgroup‐specific. Thus, they can be considered *population‐average* conditional treatment effects, and can be used to make treatment decisions for the entire population represented by this ideal RCT. When working with noncollapsible effect measures such as odds ratios or hazard ratios, conditional and marginal treatment effects do not in general coincide; conditional effects will lie further from the null.^11^ We should also be clear that the everyday usage of “conditional” to mean “depends upon” is misleading here: indeed, marginal estimates are more strongly dependent on the population than these conditional estimates, because the marginal estimates are affected by differences in all prognostic factors (observed and unobserved) whereas the conditional estimates will not be affected by differences in observed prognostic factors.^4^

R‐AHB are correct that other types of conditional effects depend on specified covariate values when treatment‐covariate interactions have been included in the analysis, and are then appropriate for decision‐making for individuals. To avoid further confusion, we refer to these conditional effects from analyses including interactions as *individual‐level* conditional treatment effects, and refer to conditional effects from analyses without interactions as *population‐average* conditional treatment effects. However, R‐AHB conflate these individual‐level and population‐average conditional treatment effects, which have different interpretations and different uses for decision making. The ML‐NMR model is parameterized in terms of individual‐level conditional treatment effects, since treatment‐covariate interactions are included in the analysis model in order to adjust for differences in effect modifiers between studies (population adjustment). However, ML‐NMR can still produce estimates of marginal and conditional population‐average treatment effects by integration over the covariate joint distribution in the target population, as we have described previously^2^, ^12^ and reiterate in the following section.

Both marginal and population‐average conditional estimands reflect a “population‐average treatment effect” as both apply to the entire population and are averaged over the distribution of covariates in the population. The marginal estimand reflects the average treatment effect over individuals in the target population without any knowledge of the distribution of prognostic covariates in the sample. The population‐average conditional estimand reflects the average treatment effect over individuals in the target population accounting for the distribution of prognostic covariates.

We consider that the *population‐average conditional treatment effect* is the most appropriate target estimand for decision makers, primarily because it reflects the recommended analysis that would be undertaken in the ideal evidence scenario described above. Decision makers typically have a well‐defined target population in mind, however, the marginal estimand does not make full use of this information. The population‐average conditional estimand is more efficient because it accounts for the known distribution of prognostic factors in the target population.^3^, ^4^, ^5^, ^6^

## 3 PRODUCING ESTIMATES OF TARGET ESTIMANDS

Although the ML‐NMR model is parameterized on individual‐level conditional treatment effects on a given linear predictor scale, ML‐NMR can produce population average estimates for a range of quantities of interest in a target population through appropriate use of integration, as described in section 2.5 of Phillippo et al.^12^

When the quantities of interest are population‐average conditional treatments effects *d*_*ab*(*P*)_ between treatments *b* and *a* in population *P*, integration simplifies to plugging in mean covariate values ${\bar{x}}_{\left(P\right)}$ in the target population since these are defined on the linear predictor scale, as given in equation (9) of our article:^2^

(1) $$\begin{array}{ll} d_{ab(P)} & =\int_{X}\left(\mu_{\left(P\right)}+x^{T}\left(\beta_{1}+\beta_{2,b}\right)+\gamma_{b}\right)f_{\left(P\right)}(x)dx-\int_{X}\left(\mu_{\left(P\right)}+x^{T}\left(\beta_{1}+\beta_{2,a}\right)+\gamma_{a}\right)f_{\left(P\right)}(x)dx\\ & ={\bar{x}}_{\left(P\right)}^{T}\left(\beta_{2,b}-\beta_{2,a}\right)+\gamma_{b}-\gamma_{a}, \end{array}$$

where $\beta_{2,b}$ and $\beta_{2,a}$ are coefficients for effect modifier interactions, and $\gamma_{b}$ and $\gamma_{a}$ are individual‐level treatment effects at the reference level of the covariates x=0. $\beta_{1}$ are coefficients for prognostic (main) effects of covariates, $\mu_{\left(P\right)}$ is a distribution for baseline response in population *P*, *f*_(*P*)_(·) is the joint covariate distribution in population *P* with support X. This does not mean that the resulting estimate is only appropriate for individuals with the mean covariate values, as suggested by R‐AHB. It just happens that the population‐average conditional treatment effect estimate on the linear predictor scale is equivalent to that for individuals with the mean covariate values.

Health economic models typically require population‐average *absolute* effects, such as average event probabilities ${\bar{p}}_{k(P)}$ on treatment *k*, which can be produced following Phillippo et al.^12^ and using the notation in our article^2^ as

(2) $${\bar{p}}_{k(P)}=\int_{X}g^{-1}\left(\mu_{\left(P\right)}+x^{T}\left(\beta_{1}+\beta_{2,k}\right)+\gamma_{k}\right)f_{\left(P\right)}(x)dx,$$

where *g*(·) is a suitable link function (eg,  logit).

Contrary to the assertion of R‐AHB that ML‐NMR cannot produce marginal population‐average treatment effect estimates, ML‐NMR can indeed estimate the marginal population‐average treatment effect $\Delta_{ab(P)}$ between treatments *b* and *a* in population *P*, simply by working with the population‐average absolute effects from (2):

(3) $$\Delta_{ab(P)}=g({\bar{p}}_{b(P)})-g({\bar{p}}_{a(P)}).$$

Estimates of other summaries of marginal population‐average treatment effects such as risk differences or relative risks can be produced by similar manipulation of ${\bar{p}}_{b(P)}$ and ${\bar{p}}_{a(P)}$. Again, we note that *d*_*ab*(*P*)_ , ${\bar{p}}_{k(P)}$, and $\Delta_{ab(P)}$ are not subgroup‐specific but apply over the entire target population *P*, since all covariates (including effect modifiers) have been integrated over.

MAIC directly targets the marginal population‐average treatment effect, and cannot estimate the (more efficient) population‐average conditional treatment effect unless suitable adjusted estimates are available from the AgD study. As described by both R‐AHB and ourselves,^2^ STC in typical usage estimates neither the marginal or conditional population‐average treatment effect and will be biased for either estimand, because in typical use STC combines conditional and marginal effects. Moreover, Equations (2) and (3) make it clear that marginal population‐average relative effects depend not only on the distribution of effect modifiers, but also on the distribution of all prognostic variables and the population baseline risk. Thus, the marginal population‐average treatment effects $\Delta_{ab(P)}$ depend more strongly on the population of interest than the population‐average conditional treatment effects *d*_*ab*(*P*)_ , and are less generalizable/transportable as a result.^4^ This is an additional concern for MAIC and STC, which produce marginal treatment effect estimates specific to the aggregate study population in a population‐adjusted indirect comparison, and may not be representative of the decision target population in either prognostic factors or effect modifiers.^13^, ^14^

## 4 COMMENT ON SIMULATION STUDY RESULTS

As we have argued above, population‐average conditional treatment effects are appropriate target estimands for efficient decision making. Our simulation study^2^ is therefore designed to evaluate the performance of the methods against the population‐average conditional treatment effects *d*_*ab*(*P*)_ —not the individual‐level conditional treatment effects $\gamma_{k}$ as R‐AHB claim.

R‐AHB suppose that much of the observed bias for MAIC in our simulation study is due to evaluating its performance against the wrong estimand, since MAIC targets the marginal population‐average treatment effect. However, standard unadjusted Bucher indirect comparisons also target marginal estimands, and yet MAIC manages to substantially increase the bias compared with these in some scenarios. Moreover, we would expect STC to perform poorly for the same reasons, since it mixes conditional and marginal estimates. However, in our simulations STC performed well and was seen to be unbiased when the requisite assumptions were met. Intuitively, therefore, our simulation scenarios must be such that the differences between marginal and population‐average conditional estimands are small. More formally, we can investigate this claim using the formula of Matthews and Badi^15^ for the ratio between the conditional and marginal estimands, which depends on the strength of the covariate effect and the variance within the population. Using this result, we determine that—even in the worst cases—the difference between marginal and population‐average conditional estimands in the scenarios we investigated is less than 0.5%. Therefore, the performance issues demonstrated for MAIC are not due to any meaningful difference in estimands, but are due to the fundamental inability of MAIC to extrapolate and the resulting bias and instability as population overlap decreases.

R‐AHB contrast our results against a simulation study of their own,^16^ which shows that MAIC can remain unbiased (for the marginal estimand) even with only moderate overlap between populations. The observed difference in performance is due to R‐AHB considering only matching covariate means across populations, whereas we consider matching both means and variances (first and second moments) which is a common approach^14^ and follows the original description of the method.^17^ The results of R‐AHB suggest that matching on covariate means only might be less sensitive to reduced population overlap and may be able to tolerate lower levels of overlap before issues arise, since this is a much less exacting requirement. This has also been observed in other simulation studies.^18^, ^19^ However, the question of when, if at all, it is preferable or necessary to match higher moments between populations for MAIC remains an interesting area for further theoretical research and simulation studies.

## 5 CONCLUSIONS

We welcome the much‐needed discussion of target estimands in the letter from R‐AHB, which has largely been overlooked in the population adjustment literature to date. We hope that our response has served to further clarify the issues surrounding estimands for noncollapsible effect measures. Here, we have argued the case for considering population‐average conditional treatment effects as appropriate targets for efficient decision making. In their letter, R‐AHB state that “methods like ML‐NMR are valid for inference at the individual level, but are not designed for population‐based inference.” However, we have demonstrated that this is not the case: ML‐NMR can indeed support inference at the individual level, but can also provide estimates of both marginal and conditional population‐average treatment effects, as well as the population‐average absolute effects typically required for health economic modeling. Moreover, ML‐NMR is likely to be a more efficient approach than MAIC even when targeting marginal population‐average treatment effects, since regression adjustment is typically more efficient than weighting.^20^ We have shown that the results of our simulation study, including the poor performance of MAIC in many scenarios, are not an artifact of noncollapsibility and incompatible estimands, and are pertinent regardless of whether population‐average conditional or marginal estimands are of interest.

R‐AHB refer to ML‐NMR as the “gold standard” for estimating conditional population‐adjusted treatment effects from mixtures of IPD and AgD, and suggest further work extending ML‐NMR to estimate marginal population‐adjusted as a research priority. However, as we have described above, ML‐NMR can be used to obtain marginal population‐adjusted treatment effects, and we would suggest therefore that ML‐NMR may also be considered the “gold standard” for estimating marginal population‐average treatment effects. Analysts and decision makers should carefully consider which target estimand is most appropriate to their needs and, as we have argued, population‐average conditional treatment effects as targeted by a hypothetical “ideal RCT” may be a more efficient choice than marginal estimands which do not account for known population characteristics.
